# Isolation and Molecular Characterization of Antimicrobial-Resistant Bacteria from Vegetable Foods

**DOI:** 10.3390/pathogens14070682

**Published:** 2025-07-10

**Authors:** Annamaria Castello, Chiara Massaro, Erine Seghers, Clelia Ferraro, Antonella Costa, Rosa Alduina, Cinzia Cardamone

**Affiliations:** 1 Experimental Zooprophylactic Institute of Sicily A. Mirri, 90129 Palermo, Italy; annamaria.castello@izssicilia.it (A.C.); cinzia.cardamone@izssicilia.it (C.C.); 2 Biological, Chemical and Pharmaceutical Sciences and Technologies Department, University of Palermo, 90129 Palermo, Italy; chiara.massaro01@community.unipa.it (C.M.); cleliaferr93@gmail.com (C.F.); 3 UFR Sciences de la Vie et de la Terre, University of Burgundy Europe, Bâtiment Gabriel, 6 Boulevard Gabriel, 21000 Dijon, France; erine.seghers@hotmail.fr

**Keywords:** antimicrobial resistance, multidrug resistance, *Enterobacteriaceae*, *Enterobacter*, *Klebsiella*, *Pseudomonas*, food safety, vegetables, one health

## Abstract

Antimicrobial resistance (AMR) poses a growing threat to global health, and its spread through the food chain is gaining increasing attention. While AMR in food of animal origin has been extensively studied, less is known about its prevalence in plant-based foods, particularly fresh and ready-to-eat (RTE) vegetables. This study investigated the occurrence of antimicrobial-resistant bacteria in fresh and RTE vegetables. Isolates were subjected to antimicrobial susceptibility testing and molecular analyses for the characterization of antimicrobial resistance genes (ARGs). A significant proportion of samples were found to harbor antimicrobial-resistant bacteria, including multidrug-resistant strains. Several ARGs, including those encoding extended-spectrum β-lactamases (ESBLs) and resistance to critically important antimicrobials, were detected. The findings point to environmental contamination—potentially originating from wastewater reuse and agricultural practices—as a likely contributor to AMR dissemination in vegetables. The presence of antimicrobial-resistant bacteria and ARGs in fresh produce raises concerns about food safety and public health. The current regulatory framework lacks specific criteria for monitoring AMR in vegetables, highlighting the urgent need for surveillance programs and risk mitigation strategies. This study contributes to a better understanding of AMR in the plant-based food sector and supports the implementation of a One Health approach to address this issue.

## 1. Introduction

The discovery of penicillin in 1928 started the golden age of antibiotics discovery, which played a key role in the reduction in the number of deaths caused by infectious diseases and the improvement of human and animal health [1]. Following a peak reached in the mid-1950s, a gradual decline in antibiotic discovery and development as well as the evolution of drug resistance have led to the current antimicrobial resistance crisis [2] when about 35,000 people die each year in the EU/EEA as a direct consequence of an infection due to antimicrobial-resistant bacteria [3]. AMR is listed among the top three priority health threats and is considered a One Health issue to be faced with a multi-sector approach and under a global perspective [4]. The Council Recommendation on stepping up EU actions to combat antimicrobial resistance in a One Health approach, adopted on 13 June 2023, highlighted that food can also play a key role in spreading AMR and exposing humans to this risk, stressing the necessity for efficient monitoring and surveillance of foodborne AMR. Foodborne AMR exposure cannot be attributed solely to food of animal origin. In fact, references suggest that the large amounts of antimicrobial residues contained in municipal wastewater [5], as well as antimicrobial-resistant bacteria and even antimicrobial resistance genes (ARGs), may not be entirely removed during the wastewater treatment process [6,7,8,9]. Therefore, once treated municipal wastewater containing these compounds is discharged to the environment, the exposure of bacteria to sublethal concentrations of antibiotics can create a selective pressure that drives the development and proliferation of antimicrobial-resistant strains. This selective pressure can be not only accidental, but also linked to the improper use of some antimicrobials in agriculture. In fact, recent references recommend the use of critically important antimicrobials for human medicine, such as amoxicillin, streptomycin, and tetracycline, in order to fight or prevent bacterial and fungal infections affecting the yield of rice, vegetable, and fruit production [10,11]. This increasing trend affects several countries among the main importers of vegetables and fruits for the EU and Italy in particular, such as the USA, China, South and Central America, and South Africa [12,13,14]. These compounds and antimicrobial-resistant bacteria can find their way into aquatic ecosystems and soil, contaminate crops, and potentially pose risks to human health through their consumption [15]. The environmental spread of antimicrobials, ARGs, and antimicrobial-resistant bacteria can affect the food safety of both fresh and ready-to-eat vegetables. In fact, the over-use of disinfectants, commonly applied also in food processing and the agricultural industry, can favor the selection of antimicrobial-resistant strains and the emergence of strains that are difficult to eradicate, hence exacerbating this widespread phenomenon [16,17]. The current regulation does not lay any criteria for food safety assessment of fresh vegetables, nor does it include the detection of antimicrobial-resistant bacteria in vegetables (both fresh and ready-to-eat) among the parameters to be assessed to ensure that these foods are safe for consumers [18].

This determines a condition where, on the one hand fruit and vegetables are reported as essential elements for a healthy lifestyle and their consumption is encouraged [19,20] and, on the other hand, there is no effective awareness of the spread of antimicrobial-resistant microorganisms among these foods, nor is there a regulated monitoring system that makes it possible to estimate the evolution of this phenomenon over the years in terms of the percentage of foods contaminated with antimicrobial-resistant microorganisms, the number and variety of antimicrobial-resistant phenotypes, and the corresponding gene determinants detected. Despite the absence of a precise and extensive monitoring system, the detection of AMR enterobacteria from these matrices is reported in various references, also recently, with resistance to ampicillin and beta-lactams in general among the most commonly mentioned [21,22]. Our investigation aimed to assess the spread of antimicrobial-resistant bacteria in fresh and ready-to-eat vegetables and characterize their antimicrobial-resistant genetic profile. This, in order to enrich the amount of data for estimation of this phenomenon, encourages the implementation and application of monitoring plans and assesses the need for measures to counteract or reduce its impact on vegetables’ food safety.

## 2. Materials and Methods

### 2.1. Sample Collection

A total of 52 vegetables intended for human consumption were analyzed. Those included n. 33 (63.46%) fresh vegetables and n. 19 (36.54%) ready-to-eat (RTE) vegetables. The fresh vegetables included n. 20 (60.61%) leafy vegetables, n. 9 (27.27%) fruit vegetables, n. 2 (6.06%) bulb vegetables, and n. 2 (6.06%) flower vegetables. The RTE vegetables included n. 17 (89.47%) leafy vegetables, n. 1 (5.26%) mixed salad containing leafy vegetables as a major component and traces of carrots, and n. 1 (5.26%) sliced carrots. They were maintained at 4 °C and analyzed within 48 h of sampling.

### 2.2. Bacteriological Analyses

The detection of *Salmonella* spp. was performed according to ISO 6579-1:2017 [23]. Presumptive colonies were screened for biochemical characterization, performed following the API 20E identification system (BioMerieux, Marcy l’Etoile, France).

The detection of *Listeria* spp. and *Listeria monocytogenes* in ready-to-eat vegetables was performed according to ISO 11290-1:2017 [24]. Presumptive colonies were confirmed by miniaturized biochemical tests (API Listeria, BioMerieux, Marcy l’Etoile, France).

β-glucuronidase-positive *Escherichia coli* enumeration was performed according to ISO 16649-2:2010 [25], and the microbial load was calculated and expressed as log CFU/g.

For *Enterococci* enumeration, samples were processed using the following self-developed method: 30 g of vegetables was diluted 1/10 (*w*/*v*) in peptone salt solution (Merk Life Science S.r.l., Milan, Italy), and serial dilutions were prepared. A total of 1 mL of each dilution was plated in rapid enterococcus agar (Oxoid, Milan, Italy) by the pour plate method. Following 44 h of incubation at 44 °C, presumptive colonies were characterized by Gram staining/microscopy (Merk Life Science S.r.l., Milan, Italy/Microscope Leica DM3000, Leica microsystems srl, Milan, Italy), a catalase test, and an esculin hydrolysis test (Esculin hydrolysis agar, Fisher scientific, Milan, Italy). Colonies were counted from plates containing 150 colonies, and the microbial load was calculated and expressed as log CFU/g.

*Enterobacteriaceae* enumeration was performed according to ISO 21528-2: 2017 [26]. Colonies were counted, and the microbial load was calculated and expressed as log CFU/g.

The microbial loads were calculated using Microsoft Excel software version 2506 Build 16.0.18925.20076 (Microsoft Corporation, Redmond, WA 98052, USA).

Following the biochemical characterization of contaminating specimens, performed by the appropriate API identification system (BioMerieux, Marcy l’Etoile, France), the antimicrobial susceptibility of the isolates was assessed by the Kirby–Bauer method (disc diffusion technique). The following 13 antimicrobials (Oxoid, Milan, Italy) were used: ampicillin (AMP; 10 μg), amoxicillin/clavulanic acid (AMC; 20 μg/10 μg), cefotaxime (CTX; 30 μg), ceftazidime (CAZ; 30 μg), kanamycin (K; 30 μg), gentamicin (CN; 10 μg), streptomycin (S; 10 μg), trimethoprim/sulfamethoxazole (STX; 25 μg), ciprofloxacin (CIP; 5 μg), nalidixic acid (NA; 30 µg), tetracycline (TE; 30 μg), chloramphenicol (C; 30 μg), and imipenem (IMP; 10 μg). The diameters of the zones of inhibition surrounding each disk were measured using a digital caliper and interpreted according to the guidelines of the Clinical Laboratory Standards Institute [27].

### 2.3. Molecular Analyses

The presence of antimicrobial resistance genes (ARGs) was screened among the strains that showed intermediate or resistant phenotypes to at least one antibiotic, as determined by the Kirby–Bauer test. The genomic bacterial DNA was extracted using the Colony PCR technique, according to Woodman et al. [28]. Briefly, 1-2 colonies from each plate were picked using a loop after overnight growth on Nutrient Agar (Oxoid, Milano, Italy). After centrifugation, the bacterial cells were dissolved in 100 μL of sterile water, vortexed for 10 s, and incubated at 99 °C for 15 min. After centrifugation at 10,000× *g* for 10 min, the supernatants were collected, and the pellets were discarded. DNA quality was assessed using 1% agarose gel electrophoresis, while purity and concentration were determined with a NanoDrop 2000c spectrophotometer (Thermo Fisher Scientific, Waltham, MA, USA). Using the primers F1 and R12 described by Coy et al. [29], the bacterial 16S rDNA gene was amplified and electrophoretically run on a 1.5% agarose gel to test the amplifiability of the extracted DNA. PCR mixes contained 2.5 μL of 10× DreamTaq Buffer (Thermo Fisher Scientific, Waltham, MA, USA), 0.5 μL of 10 pmol/μL Forward and Reverse 16S primers, 0.5 μL of 10 μM dNTPs, 0.125 μL of 5 U/μL DreamTaq DNA Polymerase (Thermo Fisher Scientific, Waltham, MA, USA), and 2 μL of DNA sample in a 25 μL total volume.

Molecular analyses to evaluate the presence of the genes related to resistance to β-lactam antibiotics were performed by Quadruplex PCR SET1 (*CTX-M IV*, *TEM, OXA*, *SHV*) and SET2 (*CMY II*, *CTX-M I*, *CTX-M II*, *DHA*), as described previously by Kim et al. [30]. We also tested the presence of specific ARGs that confer resistance to tetracyclines (*tet*A, *tet*C, *tet*D, *tet*E, *tet*G, *tet*O, *tet*W), quinolones (*qnr*A, *qnr*B, *qnr*C, *qnr*D, and *qnr*S), sulphonamides (*sul*-I, *sul*-II, *sul*-III), carbapenems (*vim*1, *vim*2, *ndm*), and cloramphenicol (*cat*1, *cat*2, *flo*R, d*hfr*1).

The PCR mixes were prepared by adding 2.5 μL of 10× DreamTaq Buffer (Thermo Fisher Scientific, Waltham, MA, USA), 0.5 μL of 20 pmol/μL μL Forward and Reverse primers (single couple or four couples of primers for Single and Quadruplex PCR, respectively), 0.5 μL of 10 μM dNTPs, 0.125 μL of 5 U/μL DreamTaq DNA Polymerase (Thermo Fisher Scientific, Waltham, MA, USA), 2 μL of DNA sample, and sterile water to a total volume of 25 µL. The thermal cycle protocol used for the PCR assays consisted of an initial step of 2 min at 95 °C followed by 35 cycles of denaturation at 95 °C for 30 s, annealing at the specific melting temperature (Tm) of each primer (as reported in Table 1) for 30 s, elongation at 72 °C for 30 s, and a final elongation step at 72 °C for 5 min.

PCR amplicons were identified by electrophoresis on 1.5% agarose gels (TAE buffer 1×), while amplicons of 120 bp were run in 1× TBE buffer on polyacrylamide gels (6% *w*/*v*), following staining with ethidium bromide and visualization under UV light.

### 2.4. Statistical Analyses

Data analysis was conducted using R Studio software version 2024.12.1+563. Spearman’s correlation analysis was performed to examine the relationship between antibiogram results and the presence or absence of antibiotic resistance genes (ARGs). The tests were two-sided, and a *p*-value lower than 0.05 was considered statistically significant.

## 3. Results

None of the 52 vegetables tested positive for *Salmonella* spp. None of the 19 RTE vegetables tested positive for *L. monocytogenes.* Referring to the enumeration of β-glucuronidase-positive *E. coli*, *Enterococci,* and *Enterobacteriaceae*, 1/52 (1.92%, fresh vegetable with microbial load 2 log CFU/g), 12/52 (23.08%) and 37/52 (71.15%), respectively, had a microbial load ≥ 1 log CFU/g (Table 2).

The following analyses were focused on *Enterobacteriaceae*. In particular, for each sample, up to four colonies with different appearance or morphology were picked from BGA (Brilliant Green Agar) and/or VRBGA (Violet Red Bile Glucose Agar) media, subjected to species identification and antimicrobial susceptibility assessment. A total of 135 strains were detected, 78 of them (57.78%) were resistant to at least one antimicrobial. Overall, 45/52 (86.54%) vegetables were found to be contaminated by at least one bacterial species resistant/intermediate resistant to at least one antimicrobial. Details about the bacterial species resistant to at least one antimicrobial and their AMR profile are listed in Table 3 and Table 4, respectively, for fresh and RTE vegetables.

As detailed in Table 5, the most widespread resistance/intermediate resistance profiles were against ampicillin (96.2%) and amoxicillin/clavulanic acid (84.6%), followed by streptomycin (38.5%), kanamycin (34.6%), and tetracycline (28.2%). The least widespread resistance/intermediate resistance profiles were against imipenem (10.3%) and ciprofloxacin (7.7%).

As shown in Table 6, among the 76 strains resistant/intermediate resistant to β-lactams subjected to molecular analyses, the most common resistance gene was *TEM* (14/76, 18.4%), followed by *CTX*-MIV (5/76, 6.6%). Three strains (3.9%) tested positive for *SHV, DHA,* and *CTX*-MI, respectively, while two strains (2.6%) had the genes *OXA* and *CMY* II. No isolates carried *CTX*-MII. Among 22 tetracycline-resistant strains, *tet*A was detected in 8 (36.4%) and *tet*W in 4 (18.2%). The genes *tet*B, *tet*C, *tet*D, and *tet*E were found in one strain each (4.5%), with no strains carrying *tet*G or *tet*O. For sulfonamide-resistant strains, 36% (4/11) carried the *sul*-I gene, and 18% (2/11) had *sul-*III. None tested positive for *sul-*II. Referring to the 22 quinolone-resistant strains, *qnr*D was detected in 7 strains (31.8%), *qnr*B in 3 strains (13.6%), *qnr*C in 2 strains (9.1%), and *qnr*S in 1 strain (4.5%). None were positive for *qnr*A.

Notably, no strains had resistance genes for carbapenems or chloramphenicol.

**Figure 1:**
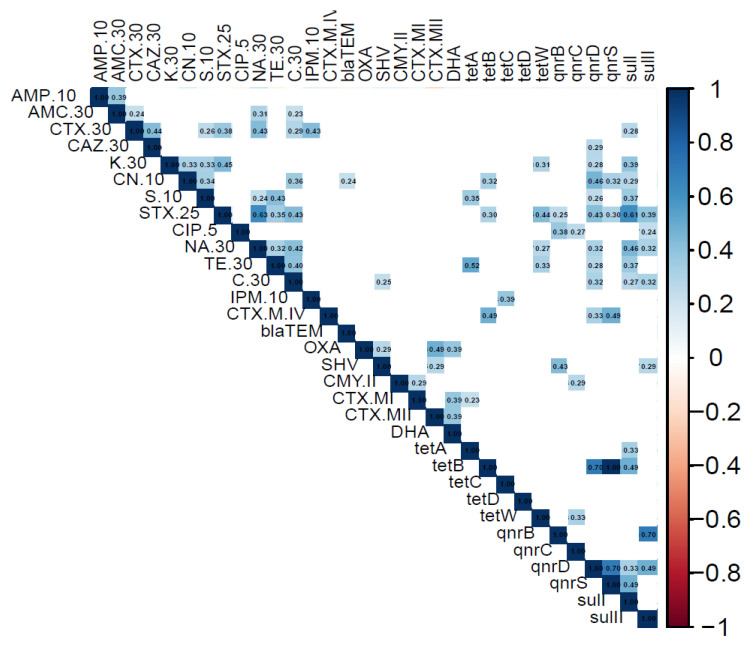
Correlation matrix of phenotypic resistance and ARGs detected. The correlation matrix and statistical Pearson test, applied to evaluate the correlation between each variable in the dataset, were created using the biostatistics R studio. The correlation matrix shows only significant (*p* < 0.05) associations. Blue: positive correlation, red: negative association, white: no correlation.

The correlation analysis (Figure 1) revealed a strong and statistically significant positive association (R = 0.61, *p*-value < 0.05) between the antibiotic trimethoprim-sulfamethoxazole (STX25) and the *sul*-I gene, indicating a link between the presence of the *sul*-I gene and phenotypic resistance to STX25. Additionally, a strong positive correlation was observed between the *qnr*D and *qnr*S genes (R = 0.73, *p*-value < 0.05), which belong to the same gene family, suggesting potential co-presence or co-regulation among the analyzed strains. Furthermore, the positive correlation between the *qnr*B and *sul-*II genes (R = 0.70, *p*-value < 0.001), as well as the correlation between the *tet*B gene and both *qnr*S and *qnr*D (R = 0.75, *p*-value < 0.01), may indicate co-localization on mobile genetic elements such as plasmids or transposons.

## 4. Discussion

The current investigation provides insight into the spread of AMR and potentially pathogenic bacteria in vegetables for human consumption. The analyses performed revealed no evidence for the causative agents of foodborne infections among the most common in edible crops, such as *L. monocytogenes* and *Salmonella* [37]. The latter has received greater attention recently due to the prolonged multi-country outbreak occurring in the EU, linked to the consumption of fresh tomatoes [38].

A high percentage of the samples analyzed (86.5%, 45/52) were found to be contaminated by AMR *Enterobacteriaceae*. These data confirm what is reported in recent references [39], suggesting that raw vegetables can be an important vehicle of AMR *Enterobacteriaceae*, which can contaminate raw vegetables through different routes, from primary production in farms to preparation in industry [40]. Once contaminated, these foods can easily vehiculate these bacteria to humans, because they are commonly consumed raw and following mild treatments. Among the isolated strains, several species commonly found in the environment and frequently associated with the emergence of AMR/MDR, opportunistic and/or nosocomial infections were identified, such as *E. cloacae* [37,41,42], *C. freundii* [43], *P. aeruginosa* [44,45], and *K. pneumoniae* [46]. Our results, together with the aforementioned references, suggest that these species might be neglected pathogens in veterinary and environmental health, and the risk of human infection concerning food consumption should be investigated more in depth [46]. The most widespread resistance/intermediate resistance profiles were recorded against ampicillin (96.2%) and amoxicillin/clavulanic acid (84.6%), followed by streptomycin (38.5%), kanamycin (34.6%), and tetracycline (28.2%). The least widespread resistance/intermediate resistance profiles were against imipenem (10.3%) and ciprofloxacin (7.7%). These results agree with recent references that report alarming data about the spread of *Enterobacteriaceae* resistant to β-lactams in vegetables, even with lower resistance percentages [40] compared to those reported here, and include carbapenems and quinolones among the still effective antimicrobials against most of the species detected [43]. In contrast, Zhang et al. [47] report sulfamethoxazole and cefotaxime resistance as the most common among various heterotrophic endophytic bacteria (HEB) isolated from crops in China, with percentages between 13% and 29%, and tetracycline and ciprofloxacin resistance as the least common (lower than 2%). Al-Kharousi et al. [21] detected 3 out of 88 isolates (3.8%) resistant to imipenem from fresh vegetables/fruits for human consumption. In this study, eight isolates showed resistance (n. 2–2.56%) or intermediate resistance (n. 6–7.69%) to imipenem. None of them carried the genes related to this phenotype searched in this work. Resistance to carbapenem can occur through different mechanisms other than the production of specific carbapenemases, such as upregulation of efflux pumps, modification of outer membrane permeability (e.g., mediated by the loss of porins), plasmid-mediated AmpC β-lactamase, and ESBL [21,48,49,50]. However, identification of mechanisms responsible for this reduced susceptibility requires further confirmation and investigation.

Even though this phenotype is generally observed in a low percentage of strains, finding reduced susceptibility to imipenem in bacteria isolated from fresh produce is significant because this antimicrobial is listed among the last available line of antibiotics, reserved for severe infections [21].

Referring to strains isolated from environmental samples, either matrices directly in contact with the environment, like crops, the incidence of AMR can be influenced by the types and amounts of molecules released in those environments where the samples are collected, which exert a selective pressure for specific antimicrobial-resistant phenotypes [51]. Other factors and conditions can affect the composition of ARGs in soil, including the typical composition of the soil and the fertilization methods applied [52]. For this reason, the incongruence between our observations and some references might be due to various aspects, including the different proportions and/or classes of selective agents most widespread in the various geographical areas covered by each study. Out of 20 MDR strains isolated, 6 belonged to the species *P. aeruginosa*, an opportunistic pathogen known for its high propensity to develop antibiotic resistance, with regard to which the emergence of multidrug-resistant strains is a major concern for global health [53].

Referring to the detection of ARGs, even though the most widespread resistance/intermediate resistance profiles were against β-lactams, only 26/76 strains carried at least one of the corresponding ARGs searched. Similarly, 6/22 tetracycline-resistant strains, 4/11 sulfonamide-resistant strains, and 17/17 chloramphenicol-resistant strains did not carry any of the corresponding ARGs searched. These data suggest that some of the AMR phenotypes revealed might be partly related to ARGs or chromosomal mutations not investigated in the present study [54,55]. Further analyses aimed at the search for additional ARGs or mutations related to AMR could provide useful data for a more comprehensive evaluation.

Considering each class of antibiotics singularly, here *bla*TEM, *tet*A, s*ul*-I, and *qnr*D were reported as the most abundant ARGs among the ones investigated for resistance to β-lactams, tetracyclines, sulphonamides, and quinolones, respectively. Our results about β-lactams resistance genes and sulphonamide resistance genes agree with recent references. In fact, *bla*TEM is often reported as the most prevalent [37,56,57] among the most abundant ARGs [22,47,58] detected in Gram-negative bacteria from vegetables, and sul-I is generally reported more frequently than *sul*-II and *sul*-III [59].

Interestingly, recent references have highlighted similarities in the prevalence levels of ARGs between vegetables and environmental samples such as water and soil. In fact, two recently published comprehensive reviews about the prevalence of ARGs in water environments report *bla*TEM, *sul*-1, and tetA genes at a high prevalence in wastewater, freshwater, seawater [60], and tap water [61], with consistent results between eastern and western countries. Also, the aforementioned genes were reported as shared between fresh fruits and soil samples by Zhang et al., 2020 [62].

Evaluating the spread of antimicrobial-resistant bacteria in fresh vegetables, fruits, and salads should be taken into great consideration, with additional attention paid to the possibility of the introduction of MDR pathogens due to international trade of fresh produce. Addressing this issue represents a key element not only for ensuring food safety, but also in a broader One Health perspective.

In fact, the extent of the spread of antimicrobial-resistant and MDR microorganisms in the aforementioned matrices can be indicative of their diffusion in the soil and water and, therefore, of environmental health and the risks associated with their transmission through routes other than the food-related ones. These risks would not involve solely humans, but all living beings that could inevitably be affected by an unequal competition with microorganisms more resistant and difficult to counteract.

From the evaluation of the current situation, the main issue emerging is a legislation gap whereby, since the detection of antimicrobial-resistant bacteria in vegetable foods is not required by the EU legislation, the current food monitoring plans do not produce the data needed for evaluating the extent of this phenomenon. The lack of data about the spread and characterization of antimicrobial-resistant and/or potentially pathogenic bacteria in such food causes the underestimation of the actual magnitude of this phenomenon; hence, the formulation of any useful initiative to limit or counteract its spread is not encouraged.

## 5. Conclusions

The spread of antimicrobial-resistant bacteria is a public health issue, resulting in increased morbidity and mortality rates. The role of vegetable foods, especially those consumed raw, as a vehicle of antimicrobial-resistant bacteria should be investigated more in-depth, extending research to different geographical areas and implementing an extensive and punctual monitoring system. The detection of antimicrobial-resistant and MDR bacterial species in vegetables is a warning sign for failure in the control of AMR. ARGs relevant to human clinical settings, as well as important opportunistic pathogens, were observed in *Enterobacteriaceae* isolated from vegetables. This demonstrates that effective control of these foods is essential for ensuring human health, but also animal health and environmental health, based on the One Health concept.

## Figures and Tables

**Table 1:** List of primers used for the PCR assays, melting temperature (Tm), amplicons expected, and references.

Target Gene	Primer Sequence (5′→3′)	Tm (°C)	Amplicon Size (bp)	Reference
*16S rDNA*	F1: GAGTTTGATCCTGGCTCAG R12: ACGGCTACCTTGTTACGACT	56	1402	[29]
*CTX-M IV*	F: GACAAAGAGAGTGCAACGGATG R: TCAGTGCGATCCAGACGAAA	61	501	[30]
*TEM*	F: AGTGCTGCCATAACCATGAGTG R: CTGACTCCCC GTCGTGTAGATA	61	431	
*OXA*	F: ATTATCTACAGCAGCGCCAGTG R: TGCATCCACGTCTTTGGTG	61	296	
*SHV*	F: GATGAACGCTTTCCCATGATG R: CGCTGTTATCGCTCATGGTAA	61	214	
*CMY II*	F: AGCGATCCGGTCACGAAATA R: CCCGTTTTATG CACCCATGA	61	695	
*CTX M I*	F: TCCAGAATAAGGAATCCCATGG R: TGCTTTACCCAGCGTCAGAT	61	621	
*CTX M II*	F: ACCGCCGATAATTCGCAGAT R: GATATCGTTGGTGGTGCCATAA	61	588	
*DHA*	F: GTGGTGGACAGCACCATTAAA R: CCTGCGGTATAGGTAGCCAGAT	61	314	
*tetA*	F: GCTACATCCTGCTTGCCTTC R: CATAGATCGCCGTGAAGAGG	60	210	[31]
*tetB*	F: TTGGTTAGGGGCAAGTTTTG R: GTAATGGGCCAATAACACCG	60	659	
*tetC*	CTTGAGAGCCTTCAACCCAG ATGGTCGTCATCTACTGCC	60	418	
*tetD*	AAACCATTACGGCATTCTGC GACCGGATACACCATCCATC	60	787	
*tetE*	F: AAACCACATCCTCCATACGC R: AAATAGGCCACAACCGTCAG	60	278	
*tetG*	F: GCTCGGTGGTATCTCTGCTC R: AGCAACAGAATCGGGAACAC	60	844	
*tetO*	F: GGAGGGGTTCAACCACAAAG R: CTATGTAAATAAAATGGATAG	55	88	
*qnrA*	F: ATTTCTCACGCCAGGATTTG R: TGCCAGGCACAGATCTTGAC	60	516	[32]
*qnrB*	F: CGACCTKAGCGGCACTGAAT R: GAGCAACGAYGCCTGGTAGYTG	50	515	
*qnrC*	F: GGGTTGTACATTTATTGAATC R: TCCACTTTACGAGGTTCT	50	446	
*qnrD*	F: CGAGATCAATTTACGGGGAATA R: AACAAGCTGAAGCGCCTG	50	581	
*qnrS*	F: GACGTGCTAACTTGCGTGAT R: TGGCATTGTTGGAAACTTG	62	118	[33]
*sul-I*	F: TCACCGAGGACTCCTTCTTC R: AATATCGGGATAGAGCGCAG	60	316	[34]
*sul-II*	F: TCCGGTGGAGGCCGGTATCTGG R: CGGGAATGCCATCTGCCTTGAG	60	191	[31]
*sul-III*	F: GAGCAAGATTTTTGGAATCG R: TCTGCAGCTAACCTAGGGCTTGGA	51	880	[34]
*vim*1	F: AGTGGTGAGTATCCGACAG R: ATGAAAGTGCGTGGAGAC	60	261	[35]
*vim*2	F: ATGTTCAAACTTTTGAGTAAG R: CTACTCAACGACTGAGCG	60	801	
*ndm*	F: GGTTTGGCGATCTGGTTTTC R: CGGAATGGCTCATCACGATC	60	621	
*imp*1	F: CTACCGCAGCAGAGTCTTTG R: AACCAGTTTTGCCTTACCAT.	55	587	
*imp*2	F: GTTTTATGTGTATGCTTCC R: AGCCTGTTCCCATGTAC	55	678	
*cat-*1	F: CCTATAACCAGACCGTTCAG R: TCACAGACGGCATGATGAAC	56	495	[36]
*cat-*2	F: CCGGATTGACCTGAATACCT R: TCACATACTGCATGATGAAC	56	572	
*floR*	F: AACCCGCCCTCTGGATCAAGTCAA R: CAAATCACGGGCCACGCTGTATC	60	548	
*dhfr* 1	F: GTGAAACTATCACTAATGGTAGCT R: ACCCTTTTGCCAGATTTGGTAACT	54	470	

**Table 2:** Data about the samples with microbial loads ≥ 1 log CFU/g detected for *Enterococci* and *Enterobacteriaceae*.

Enterococci			Enterobacteriaceae		
	No. of Samples	M ± SD ^2^		n. of Samples	M ± SD ^2^
Fresh vegetables	11	3.44 ± 1.28	Fresh vegetables	23	3.09 ± 1.09
RTE ^1^ vegetable	1	1	RTE ^1^ vegetable	14	3.29 ± 1.22
Total	12	3.24 ± 1.41		37	3.17 ± 1.21

^1^ ready-to-eat, ^2^ media ± standard deviation.

**Table 3:** Bacterial species resistant to at least 1 antimicrobial detected in fresh vegetables.

Strain ID	Species	AMR Profile	
		R	I
1	*E.* ^1^ *cloacae*	AMP10, AMC30	
2	*E.* ^2^ *coli*	AMP10, AMC30, TE30	
3	*H.* ^3^ *alvei*	AMP10, AMC30	CAZ30
4	*H.* ^3^ *alvei*	AMP10, AMC30	CAZ30
5	*E.* ^1^ *cloacae*	AMP10, AMC30	
6	*E.* ^1^ *cloacae*	AMP10, AMC30, STX25, TE30, C30	K30, NA30
7	*P.* ^4^ *fluorescens*	AMP10, AMC30, CTX30, NA30, TE30, C30, IPM10	
8	*Acinetobacter*	AMP10, AMC30	
10	*P.* ^4^ *aeruginosa*	AMP10, AMC30, K30, CN10, S10, STX25, NA30, TE30, C30	
11	*C.* ^5^ *freundii*	AMP10, AMC30	
12	*E.* ^1^ *cloacae*	AMP10, AMC30	
13	*C.* ^5^ *freundii*	AMP10, AMC30	
14	*M.* ^6^ *morganii*	AMP10, AMC30, TE30, C30	
15	*P.* ^4^ *aeruginosa*	AMP10, AMC30, K30, CN10, S10, STX25, NA30, TE30, C30	CTX30, CAZ30
16	*E.* ^1^ *cloacae*	AMP10	AMC30
17	*C.* ^5^ *freundii*	AMP10, AMC30	
18	*P.* ^7^ *rettgeri*	AMC30, TE30	
19	*C.* ^5^ *freundii*	AMP10, AMC30	
20	*P.* ^4^ *aeruginosa*	AMP10, AMC30, K30, CN10, S10, STX25, NA30, TE30, C30	CTX30, CAZ30
21	*E.* ^1^ *cloacae*	AMP10, AMC30	
22	*E.* ^1^ *cloacae*	AMP10, AMC30	
23	*K.* ^8^ *pneumoniae*	AMP10, K30, CN10, S10, TE30	
24	*E.* ^1^ *cloacae*	AMP10, AMC30, CN10	K30, S10, TE30, C30
26	*K.* ^8^ *oxytoca*	AMP10, S10	K30, CN10
27	*Citrobacter*	K30	AMP10
28	*K.* ^8^ *pneumoniae*	AMP10, AMC30	S10
29	*Enterobacter* (EMP)	AMP10, K30	S10
30	*K.* ^8^ *pneumoniae*	AMP10	S10
31	*E.* ^1^ *cloacae*		AMP10, AMC30, K30, S10
35	*P.* ^7^ *stuartii*	AMP10, AMC30, S30, TE30	K30
36	*E.* ^1^ *cloacae*	AMP10, AMC30, S30, TE30	
38	*E.* ^1^ *cloacae*	AMP10, AMC30, NA30, C30	STX25, CIP5
49	*P.* ^4^ *fluorescens*	AMP10, AMC30, CTX30, STX25, NA30	
50	*E.* ^1^ *cloacae*	AMP10, AMC30, CTX30, CN10	C30
51	*K.* ^8^ *pneumoniae* spp. *ozaenae*	CTX30	AMP10, AMC30, CN10, C30
52	*E.* ^1^ *cloacae*	AMP10, AMC30, CN10	
53	*E.* ^1^ *cloacae*	AMP10, AMC30, CTX30, NA30	S10, IPM10
54	*E.* ^1^ *cloacae*	AMP10, AMC30, CN10; I: CTX30, K30, IPM10	
56	*P.* ^4^ *aeuruginosa*	AMP10, AMC30, CTX30, K30, CN10, STX25, NA30	TE30
57	*R.* ^9^ *ornithlytica*	AMP10, AMC30, CTX30, CAZ30, S10, CIP 5, C30	NA30
58	*Cronobacter* spp.	R: AMP10, AMC30, CTX30, S10, C30, IPM10	CAZ30, K30, CN10, NA30
59	*Pantoea* spp.	K30, STX25, CIP 5, C30	AMP10, AMC30, CTX30, CAZ30
60	*K.* ^8^ *oxytoca*	AMP10, AMC30, K30	S10
61	*A.* ^10^ *hydrophila*	AMP10, AMC30, K30	CIP 5
62	*C.* ^5^ *freundii*	AMP10, AMC30, S10	CIP 5
65	*R.* ^11^ *pickettii*	AMP10, AMC30	NA30, C30
67	*E.* ^1^ *cloacae*	AMP10, AMC30; I: K30, S10, IPM10	
68	*P.* ^7^ *rettgerii*	AMC30, TE30	CAZ30, S10
69	*E.* ^1^ *cloacae*	AMP10, AMC30	K30, TE30
70	*E.* ^3^ *coli type 1*	AMP10, AMC30, S10	NA30, TE30
75	*K.* ^8^ *pneumoniae* spp. *pneumoniae*	AMP10, AMC30, C30	CAZ30

^1^ *Enterobacter*; ^2^ *Hafnia*; ^3^ *Escherichia*; ^4^ *Pseudomonas*; ^5^ *Citrobacter*; ^6^ *Morganella*; ^7^ *Providencia*; ^8^ *Klebsiella*; ^9^ *Roultella*; ^10^ *Aeromonas*; ^11^ *Ralstonia*. R: resistant, I: intermediate resistant.

**Table 4:** Bacterial species resistant to at least 1 antimicrobial detected in RTE vegetables.

Strain ID	Species	AMR Profile	
		R	I
37	*E.* ^1^ *cloacae*	AMP10, AMC30	
39	*R.* ^2^ *ornithinolytica*	AMP10	
40	*C.* ^3^ *freundii*	AMP10	AMC30
41	*Pantoea* spp.	AMP10	
42	*P.* ^4^ *fluorescens*	AMP10, AMC30, NA30	CAZ30
43	*K.* ^5^ *pneumoniae* spp. *ozaenae*	AMP10, K30	
44	*K.* ^5^ *oxytoca*	AMP10	
45	*C.* ^3^ *youngae*	AMP10, K30	
46	*E.* ^6^ *coli*	AMP10	
47	*R.* ^2^ *aquatilis*	AMP10, K30	
48	*K.* ^5^ *pneumoniae* spp. *pneumoniae*	AMP10, AMC30, K30	S10
55	*E.* ^1^ *cloacae*	AMP10, AMC30, TE30	CTX30, CAZ30, S10, C30, IPM10
63	*E.* ^6^ *coli tipo 1*	AMP10, S10	AMC30, CTX30, CAZ30
64	*M.* ^7^ *morganii*	AMP10, AMC30	CTX30, K30, S10, IPM10
66	*K.* ^5^ *pneumoniae*	S10	AMP10, AMC30
71	*P.* ^4^ *aeuruginosa*	AMP10, AMC30, CTX30, K30, S10, STX25, NA30, TE30	C30
72	*E.* ^1^ *cloacae*	AMP10, AMC30, S10, CIP5	CTX30
73	*C.* ^3^ *youngae*	AMP10, S10, C30	AMC30, TE30
74	*C.* ^3^ *youngae*	C30	AMC30, TE30
76	*C.* ^3^ *freundii*	AMP10, AMC30, CAZ30, NA30, TE30	S10
77	*E.* ^1^ *cloacae*	AMP10, AMC30	
78	*A.* ^8^ *hydrophila*	AMP10	AMC30, CAZ30, K30
79	*E.* ^1^ *cloacae*	AMP10, AMC30	
80	*H.* ^9^ *alvei*	AMP10, AMC30, CTX30, CAZ30	
81	*E.* ^1^ *cloacae*	AMP10, AMC30, CAZ30	CTX30, IPM10
82	*P.* ^4^ *aeruginosa*	AMP10, AMC30, K30, STX25, NA30	CTX30, S10, TE30
83	*K.* ^5^ *oxytoca*	AMP10	AMC30, NA30

^1^ *Enterobacter*; ^2^ *Roultella*; ^3^ *Citrobacter*; ^4^ *Pseudomonas*; ^5^ *Klebsiella*; ^6^ *Escherichia*; ^7^ *Morganella*; ^8^ *Aeromonas*; ^9^ *Hafnia*. R: resistant, I: intermediate resistant.

**Table 5:** Percentages of resistance, intermediate resistance, and susceptibility revealed against each antimicrobial.

	AMP	AMC	CTX	CAZ	K	CN	S	STX	CIP	NA	TE	C	IPM
R (%)	88.5	69.2	12.8	6.4	20.5	11.5	20.5	11.5	3.8	15.4	19.2	16.7	2.6
I (%)	7.7	15.4	12.8	15.4	14.1	3.9	18.0	1.3	3.9	7.7	9.0	7.7	7.7
S (%)	3.8	15.4	74.4	78.2	65.4	84.6	61.5	87.2	92.3	76.9	71.8	75.6	89.7

AMP: ampicillin, AMC: amoxicillin/clavulanic acid, CTX: cefotaxime, CAZ: ceftazidime, K: kanamycin, CN: gentamicin, S: streptomycin, STX: trimethoprim/sulfamethoxazole, CIP: ciprofloxacin, NA: nalidixic acid, TE: tetracycline, C: chloramphenicol, IMP: imipenem.

**Table 6:** Distribution of ARGs among the seventy-six strains screened.

	GENE	N	%
β-lactams	*TEM*	14/76	18.4%
	*CTX-*M IV	5/76	6.6%
	*SHV*	3/76	3.9%
	*DHA*	3/76	3.9%
	*CTX*-MI	3/76	3.9%
	*CMY*-II	2/76	2.6%
	*OXA*	2/76	2.6%
	*CTX*-MII	0/76	0%
Tetracyclines	*tet*A	8/22	36.4%
	*tet*W	4/22	18.2%
	*tet*B	1/22	4.5%
	*tet*C	1/22	4.5%
	*tet*D	1/22	4.5%
	*tet*E	1/22	4.5%
	*tet*G	0/22	0%
	*tet*O	0/22	0%
Sulphonamides	*sul-*I	4/11	36%
	*sul-*II	0/11	0%
	*sul-*III	2/11	18%
Quinolones	*qnr*D	7/22	31.8%
	*qnr*B	3/22	13.6%
	*qnr*C	2/22	9.1%
	*qnr*S	1/22	4.5%
	*qnr*A	0/22	0%
Carbapenems	*vim*1	0/7	0%
	*vim*2	0/7	0%
	*ndm*	0/7	0%
	*imp*1	0/17	0%
	*imp*2	0/17	0%
Chloramphenicol	*cat*1	0/17	0%
	*cat*2	0/17	0%
	*flo*R	0/17	0%
	*dflo*R	0/17	0%

## Data Availability

The original contributions presented in this study are included in the article. Further inquiries can be directed to the corresponding authors.
